# Contributions of K_ATP_ and K_Ca_ channels to cerebral arteriolar dilation to hypercapnia in neonatal brain

**DOI:** 10.14814/phy2.12127

**Published:** 2014-08-28

**Authors:** Chukwuma C. Nnorom, Corinne Davis, Alexander L. Fedinec, Khadesia Howell, Jonathan H. Jaggar, Helena Parfenova, Massroor Pourcyrous, Charles W. Leffler

**Affiliations:** 1 Departments of Physiology and Pediatrics, University of Tennessee Health Science Center, Memphis, Tennessee

**Keywords:** Cerebrovascular circulation, in vivo cranial window, newborn pig, potassium channels

## Abstract

Mechanisms by which Pco_2_ controls cerebral vascular tone remain uncertain. We hypothesize that potassium channel activation contributes to the neonatal cerebrovascular dilation in response to increases in Paco_2_. To test this hypothesis, experiments were performed on newborn pigs with surgically implanted, closed cranial windows. Hypercapnia was induced by ventilation with elevated Pco_2_ gas in the absence and presence of the K_ATP_ channel inhibitor, glibenclamide and/or the K_Ca_ channel inhibitor, paxillin. Dilations to pinacidil, a selective K_ATP_ channel activator, without and with glibenclamide, were used to evaluate the efficacy of K_ATP_ channel inhibition. Dilations to NS1619, a selective K_Ca_ channel activator, without and with paxillin, were used to evaluate the efficacy of K_Ca_ channel inhibition. Cerebrovascular responses to the K_ATP_ and K_Ca_ channel activators, pinacidil and NS1619, respectively, cAMP‐dependent dilator, isoproterenol, and cGMP‐dependent dilator, sodium nitroprusside (SNP), were used to evaluate the selectivity of glibenclamide and paxillin. Glibenclamide blocked dilation to pinacidil, but did not inhibit dilations to NS1619, isoproterenol, or SNP. Glibenclamide prior to hypercapnia decreased mean pial arteriole dilation ~60%. Glibenclamide treatment during hypercapnia constricted arterioles ~35%. The level of hypercapnia, Paco_2_ between 50 and 75 mmHg, did not appear to be involved in efficacy of glibenclamide in blocking dilation to Paco_2_. Similarly to glibenclamide and K_ATP_ channel inhibition, paxillin blocked dilation to the K_Ca_ channel agonist, NS1619, and attenuated, but did not block, arteriolar dilation to hypercapnia. Treatment with both glibenclamide and paxillin abolished dilation to hypercapnia. Therefore, either glibenclamide or paxillin that block dilation to their channel agonists, pinacidil or NS1619, respectively, only partially inhibit dilation to hypercapnia. Block of both K_ATP_ and K_Ca_ channels completely prevent dilation hypercapnia. These data suggest hypercapnia activates both K_ATP_ and K_Ca_ channels leading to cerebral arteriolar dilation in newborn pigs.

## Abstract (precis)

Mechanisms by which Pco_2_ controls vascular tone remain uncertain. We hypothesize K_ATP_ and K_Ca_ channel activation contributes to the neonatal cerebrovascular dilation in response to increases in Paco_2_. Presented data support this hypothesis.

## Introduction

Disorders of the cerebral circulation are a prominent cause of mortality and morbidity in newborns and can result in lifelong disabilities (Du Plessis and Volpe 2002; Shankaran and Laptook 2007; Dehaes et al. 2014). The neonatal brain is more susceptible to injury from fluctuations in blood flow than the adult brain because of rapid development and proliferation of new neurons and vessels (Calvert and Zhang 2005). A better understanding of cerebrovascular control mechanisms is thus vital to neonatal medicine.

There is no question that regulatory mechanisms of cerebral control are not the same as those in the adult (Leffler 1997; Fermandez‐Lopez et al. 2014). To understand the physiology and pathophysiology, we must study newborns. Newborn pigs are an outstanding species to use because of virtually identical cardiovascular system, rapid maturation, and size that allows many techniques impossible in a newborn rodent. For example, cerebral circulatory prostacyclin dominance switches over to NO over weeks to months (Willis and Leffler 1999).

In the cerebral vasculature, arterial and arteriolar partial pressure of CO_2_ (Paco_2_) is a primary regulatory stimulus controlling blood flow (Busija and Heistad 1984). The mechanisms involved in cerebrovascular tone regulation by Paco_2_ in the newborn are of clinical significance, in part, because current clinical practice in mechanical ventilation of preterm newborns is to permit higher Paco_2_ by using lower ventilator pressures with the goal of preventing lung injury (Calvert and Zhang 2005; Croinin et al. 2005; Kavanagh and Laffey 2006). The resulting elevation of Paco_2_ above normal dilates cerebral arterioles.

ATP‐sensitive potassium (K_ATP_) channels have been shown to be present in the cerebral vasculature of some animal species (Busija and Heistad 1984; Masuzawa et al. 1990). In adult rat systemic arteries, K_ATP_ channel activation appears to be involved in dilation caused by hypercapnia (Masuzawa et al. 1990; Wang et al. 2003; Zhuo et al. 2005). Also, in a recent publication, we showed K_ATP_ channel activation as a mechanism for H_2_S‐induced dilation in newborn piglet cerebral arterioles (Leffler et al. 2011). This is relevant because inhibition of H_2_S production decreased pial arteriolar dilation to hypercapnia. These combined findings suggest there may be a role of K_ATP_ channels in hypercapnia‐induced cerebrovascular dilation in newborns.

However, the participation of K_ATP_ channels in the neonatal cerebrovascular response to hypercapnia remains uncertain (Leffler et al. 1997).

Therefore, the present study was initially designed to address the hypothesis that K_ATP_ channels play a role in neonatal cerebral arteriolar vasodilation in response to hypercapnia.

## Methods

All procedures involving animals have been reviewed and approved by the Animal Care and Use Committee of the University of Tennessee Health Science Center. Newborn pigs (within the first 7 days of life) weighing 1.5–3 kg were anesthetized with ketamine hydrochloride (33 mg/kg, i.m.) and acepromazine (3.3 mg/kg, i.m.) or ketamine hydrochloride (15 mg/kg, i.m.) and xylazine (2 mg/kg, i.m.). We have been unable to detect any effect of the preanesthetic on subsequent cerebrovascular responses to any vasogenic stimulus tested. Anesthesia was maintained by *α*‐chloralose (50 mg/kg, i.v.).

The piglets were intubated via tracheostomies and placed on mechanical ventilation. Femoral veins were cannulated for anesthetic injection and femoral arteries were cannulated for continuous blood pressure monitoring and arterial blood sampling for blood gas and pH measurements. Blood pressure, blood gases, pH, and body temperature were maintained within normal range except during hypercapnia treatment.

### Cranial window implantation

The scalp of each piglet was incised and retracted, and an opening of 2 cm in diameter created through the skull over the parietal cortex. The dura mater was cut and then placed over the cut bone edges. A stainless steel frame with a glass pane was placed in the hole, sealed with bone wax, and fixed with dental acrylic. The cranial window frame had side needle ports through which artificial cerebrospinal fluid (aCSF) was placed under the window. The aCSF was equilibrated with 6% CO_2_‐ and 6% O_2_‐producing gases and pH within normal range for CSF (pH = 7.35–7.40) and both Po_2_ and Pco_2_ between 42 and 46 mmHg. Pial vessels were observed through the window with a dissecting microscope. Arteriole diameters were measured using a video micrometer coupled to a television camera mounted on the microscope and a video monitor.

### Pharmacological agents used

All pharmacological agents were applied topically in the aCSF under the window. Pinacidil (10^−5^ mol/L) was used as the most selective activator of K_ATP_ channels. NS1619 (2 × 10^−6^ mol/L), a K_Ca_ channel agonist, was used as a control that dilates cerebral arterioles by a K_ATP_ channel‐independent mechanism. Similarly, sodium nitroprusside (SNP) (10^−6^ mol/L) and isoproterenol (10^−6^ mol/L) were used as endothelium‐independent, cGMP‐ and cAMP‐dependent vasodilators, respectively. Dilations to pinacidil, NS1619, SNP, and/or isoproterenol were measured before and in the presence of the potent and selective K_ATP_ channel antagonist glibenclamide (10^−7^ or 10^−6^ mol/L) as described in the text. Artificial CSF without or with glibenclamide, as appropriate, was used to flush the windows (as controls) between experimental treatments and allowing the pial arteriolar diameter to return to baseline.

### Dilation to hypercapnia

Hypercapnia was caused by ventilation with 5% or 10% CO_2_, 21% O_2_, and the balance N_2_. These mixtures increased the Paco_2_ to approximately 50 and 75 mmHg, respectively, from the baseline Paco_2_ of 30–40 mmHg. As described in Results, in some piglets, we measured dilation to hypercapnia before and then in the presence of glibenclamide to determine if K_ATP_ channel blockade can prevent dilation to hypercapnia. In others, glibenclamide was placed under the cranial window at 5 min of hypercapnia and pial arteriolar diameters were recorded at 5, 10, and 15 min after glibenclamide during hypercapnia to see if inhibition of K_ATP_ channels can reverse dilation to hypercapnia.

The other pharmacological agents described above were used to confirm efficacy and selectivity of glibenclamide or paxillin as an inhibitor of K_ATP_ channels and K_Ca_ channels, respectively.

Glibenclamide was stored in ethanol (10^−3^ mol/L, −20°C) or dissolved freshly in DMSO and diluted with aCSF to 10^−7^ or 10^−6^ mol/L for injection under the cranial window. The blockade of dilation to pinacidil (10^−5^ mol/L) by glibenclamide confirmed the K_ATP_ channels were blocked and no change to isoproterenol (10^−6^ mol/L), SNP (10^−5^ mol/L), or NS1619 (10^−6^ mol/L) confirmed inhibition is selective for K_ATP_ channels. Time‐vehicle controls (repeat without glibenclamide or paxillin) were done randomly with the protocols without apparent or significant differences. Protocols for paxillin were the same with the anticipated and produced result of inhibition of dilation to paxillin being NS1619 rather than pinacidil.

### Statistical analysis

Data are presented as means ± SEM. Comparison among populations within each experimental group used ANOVA with or without repeated measures depending on experimental design, followed by Tukey post hoc test to determine differences between groups. *P* < 0.05 was considered significant.

## Results

**Figure 1.**
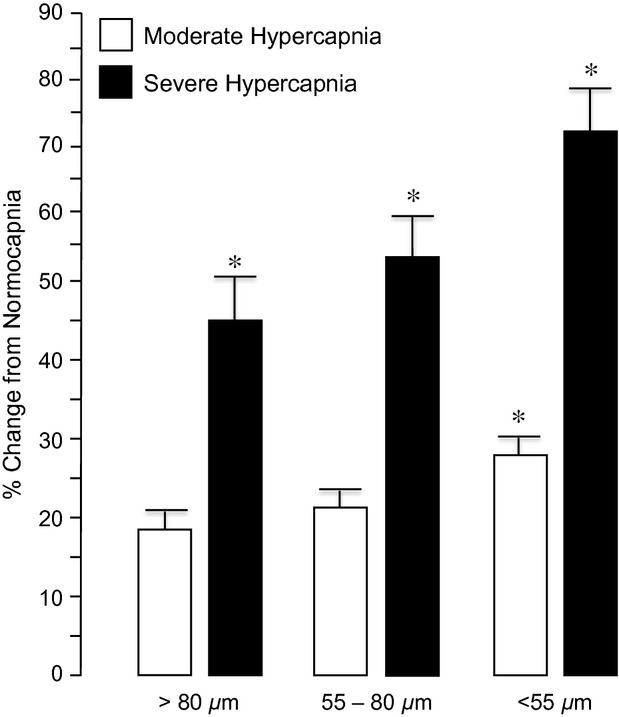
Effect of arteriole size on dilation to moderate hypercapnia (Paco_2_ ~54 mmHg) and severe hypercapnia (Paco_2_ ~87 mmHg) (see Table 1). *n* = 8 piglets. *P < 0.05 compared to normocapnia (Paco_2_ ~35 mmHg).

**Figure 2.**
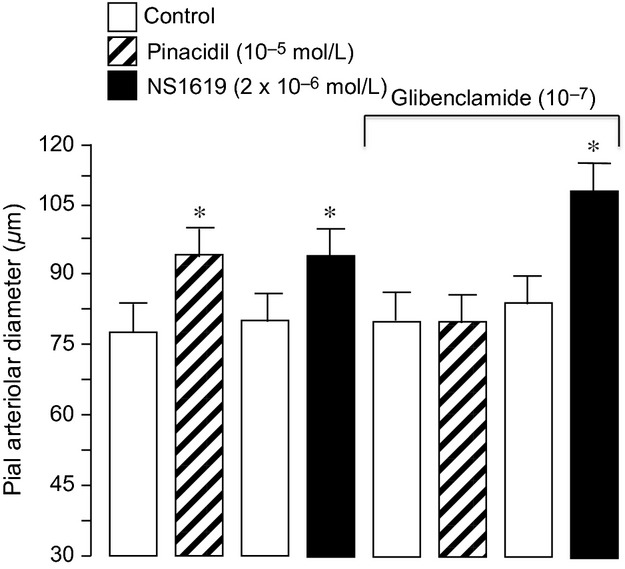
The effect of pinacidil and NS1619 on pial arteriolar diameter of newborn pigs before and in the presence of glibenclamide (10^−7^ mol/L). Mean ± SEM,* n* = 8 piglets. *P < 0.05 compared to preceding control.

**Figure 3.**
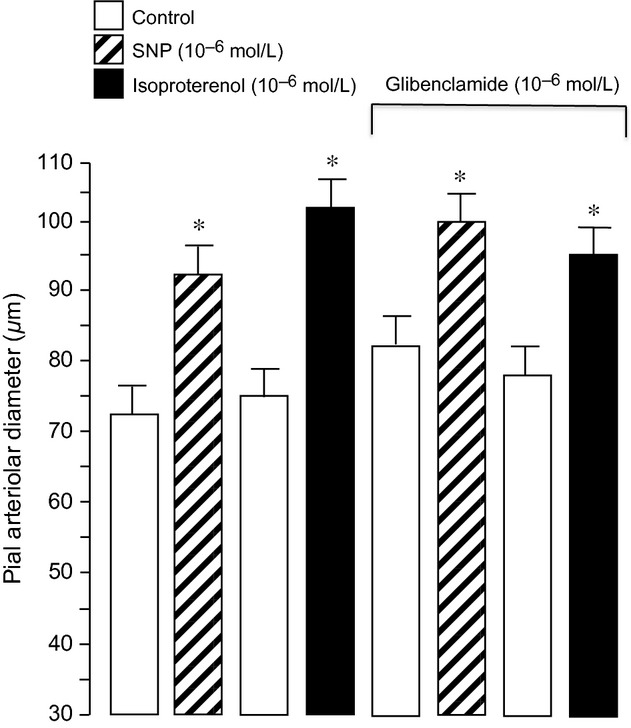
The effect of sodium nitroprusside (SNP) and isoproterenol on pial arteriolar diameter of newborn pigs before and in the presence of glibenclamide (10^−7^ mol/L). Mean ± SEM,* n* = 6 piglets. *P < 0.05 compared to preceding control.

Dilation of newborn pial arterioles to hypercapnia may be dependent, in part, to the size of the vessels measured. Therefore, we divided the control arterioles into three groups: large arterioles (>80 *μ*m), medium arterioles (55–80 *μ*m), and small arterioles (less than 55 *μ*m) (Fig. 1). In this report, the average and median arteriole had a diameter of ~70 *μ*m. As expected, pial arterioles of all sizes dose‐dependently dilated in response to hypercapnia. The dilations increased progressively as the size of the arteriole decreased between 80 and 40 *μ*m. Topical application of pinacidil (10^−5^ mol/L), a K_ATP_ channel activator, caused pial arteriolar dilation in newborn pigs (Fig. 2). This dilation was blocked by glibenclamide placed under the cranial window, demonstrating efficacy of K_ATP_ channel inhibition. In contrast, glibenclamide alone had no effect on newborn piglet pial arteriolar diameters (PAD) (Fig. 2). Also, dilation to NS1619, a K_Ca_ channel activator, was not inhibited by glibenclamide (Fig. 2). Dilation to sodium nitroprusside (SNP) and isoproterenol, that increase cGMP and cAMP, respectively, also cause dilation that is not blocked by glibenclamide (Fig. 3).

Table 1 shows blood gases, pH, and mean arterial pressure during ventilation with air, “moderate hypercapnia,” and “severe hypercapnia.” This elevated Paco_2_ gas is produced by 5 min ventilation with half (moderate hypercapnia) or all (severe hypercapnia) with 10% CO_2_, 20% O_2_, balance nitrogen (Table 1). Table 1 shows a pure respiratory acidosis. There was a small drop in mean arterial pressure, presumably resulting from massive systemic vasodilation that the newborn heart cannot match.

**Table 1.** Effects of ventilation with elevated Pico_2_ on arterial blood gases, pH, and mean arterial pressure (MAP) of newborn pigs (*n* = 36)

	Normocapnia	Moderate hypercapnia	Severe hypercapnia
Pao_2_ (mmHg)	111.7 ± 2.1	103.6 ± 2.2*	94.6 ± 1.9*
Paco_2_ (mmHg)	35.0 ± 0.95	53.9 ± 0.88*	87.1 ± 1.7*
pH_a_	7.46 ± 0.01	7.28 ± 0.06*	7.09 ± 0.02*
MAP (mmHg)	72.0 ± 1.5	72.2 ± 1.8	64 ± 2.2*

*P < 0.05 compared to normocapnia.

**Figure 4.**
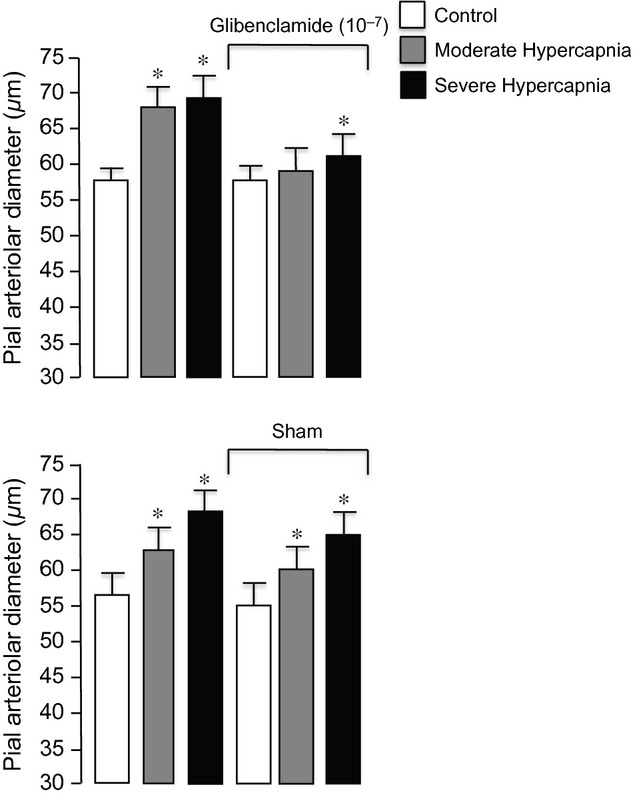
The effect of glibenclamide on dilation to moderate hypercapnia (Paco_2_ ~50 mmHg) and severe hypercapnia (Paco_2_ ~70 mmHg). Mean ± SEM,* n* = 6 piglets each, glibenclamide and control. *P < 0.05 compared to normocapnia.

Piglets that were ventilated with air, moderate, and then severe hypercapnia twice had very similar dilations to hypercapnia (Fig. 4, bottom panel). Conversely, cerebrovascular dilation to hypercapnia was inhibited by topical application of glibenclamide (Fig. 4, top).

**Figure 5.**
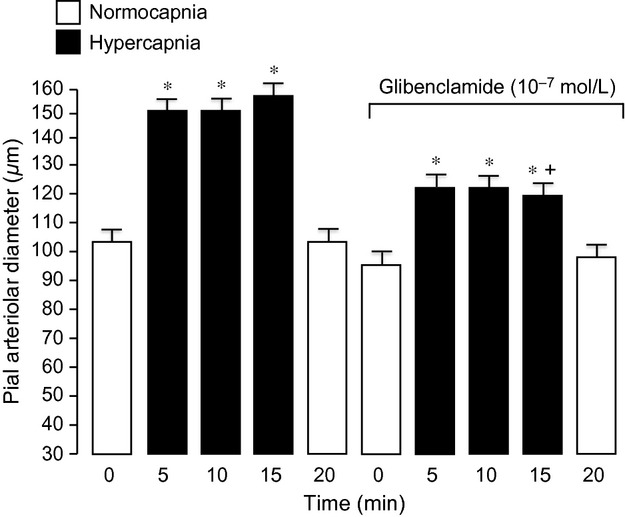
The effect of glibenclamide on dilation to 15‐min hypercapnia (Paco_2_ ~70 mmHg). Mean ± SEM,* n* = 8 piglets. *P < 0.05 compared to previous normocapnia. ^†^*P* < 0.05 compared to before glibenclamide.

**Figure 6.**
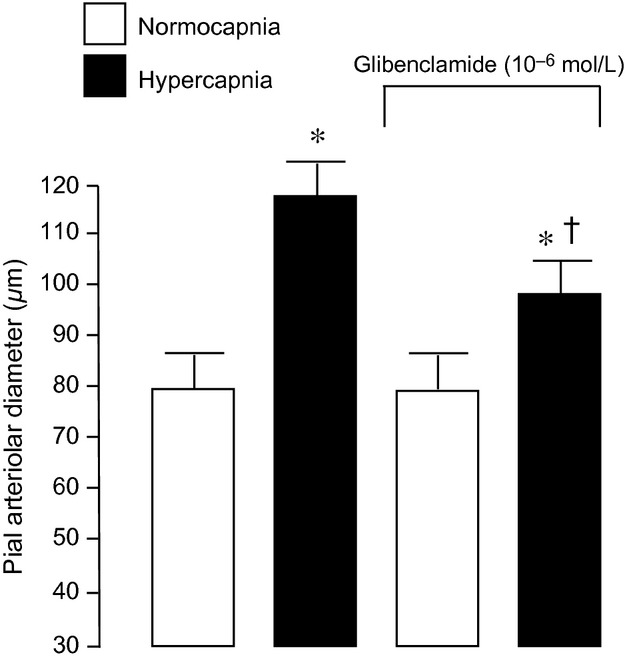
The effect of glibenclamide (10^−6^ mol/L) on pial arteriolar dilation to hypercapnia before and after glibenclamide pretreatment. Mean ± SEM,* n* = 6 piglets. *P < 0.05 compared to normocapnia. ^†^*P* < 0.05 compared to hypercapnia without glibenclamide.

Ventilation of piglets with 10% CO_2_ increased Paco_2_ to 70–80 mmHg within 5 min and caused a rapid, sustained, and reversible dilation of pial arterioles (Table 1, Fig. 5). Glibenclamide (10^−7^ mol/L) reduced the dilation to hypercapnia (Fig. 5). Increasing the glibenclamide concentration to 10^−6^ mol/L did not increase the inhibition further (Fig. 6).

**Figure 7.**
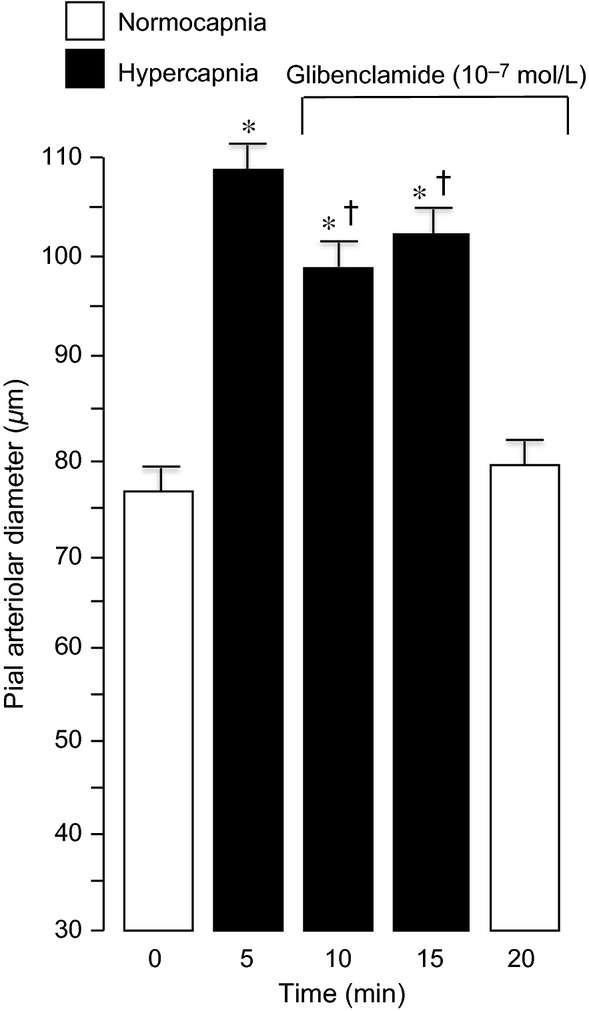
Effects of glibenclamide (10^−7^ mol/L) on pial arteriolar diameter beginning 5 min after the onset of hypercapnia. Mean ± SEM,* n* = 14 piglets. *P < 0.05 compared to normocapnia. ^†^*P* < 0.05 compared to hypercapnia without glibenclamide.

**Figure 8.**
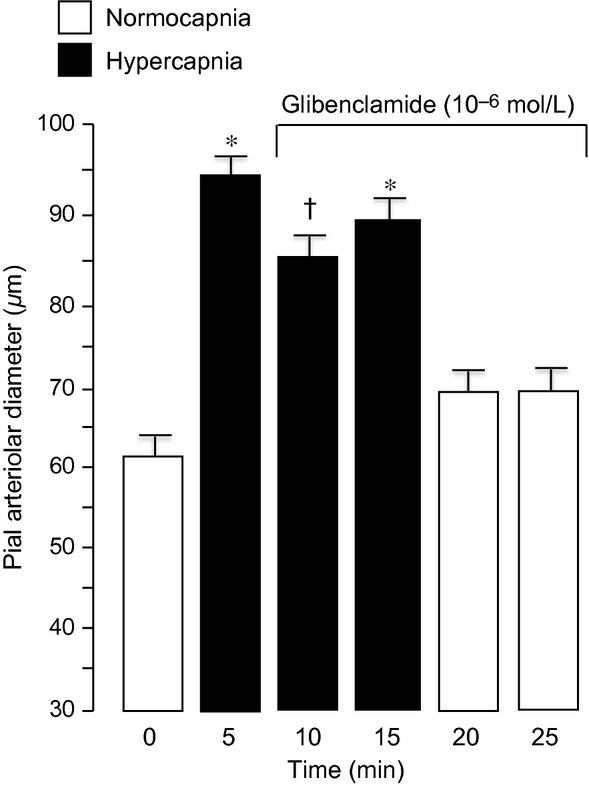
Effects of glibenclamide (10^−6^ mol/L) (on pial arteriolar diameter beginning 5 min after the onset of hypercapnia. Mean ± SEM,* n* = 6 piglets. *P < 0.05 compared to normocapnia. ^†^*P* < 0.05 compared to hypercapnia without glibenclamide.

Topical application of glibenclamide (10^−7^ mol/L) under the cranial window when hypercapnia‐induced dilation is already established caused a constriction of about 35% (Fig. 7). Constriction caused by 10‐fold higher glibenclamide was not markedly increased (Fig. 8).

From the above, it is clear that glibenclamide – that blocks dilation to pinacidil completely – inhibits but does not block dilation to hypercapnia. In search of the signal that causes the rest of the dilation to hypercapnia, we hypothesized that large conductance K_Ca_ channels also contribute to control of newborn cerebral arterioles in response to hypercapnia. Our data suggest that hypercapnia stimulates K_Ca_ channels causing arteriolar hyperpolarization and dilation in addition to the K_ATP_ channels.

**Figure 9.**
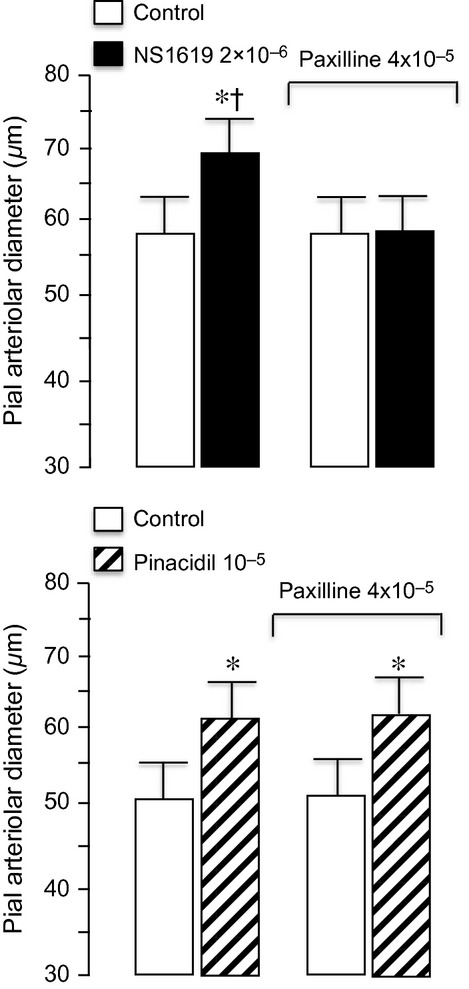
Effects of paxillin on dilation of newborn cerebral arterioles in vivo. Topical paxillin (4 × 10^−5^ mol/L) completely blocked pial arteriolar dilation to the K_Ca_ channel activator, NS1619, top panel (*n* = 5 piglets), but had no effect on dilation to the K_ATP_ channel activator, pinacidil (*n* = 3 piglets). *P < 0.05 compared to the preceding control. ^†^*P* < 0.05 compared to pinacidil and paxillin.

**Figure 10.**
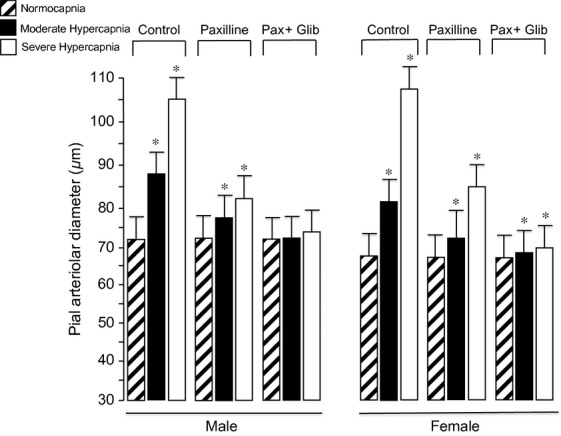
Effects of paxillin (4 × 10^−5^ mol/L) (Pax) alone or in combination with glibenclamide (10^−7^ mol/L) (Glib) on hypercapnia‐induced cerebrovascular dilation in both genders. *n* = 8 piglets.*P < 0.05 compared to the preceding normocapnia. *n* = 8 of each gender.

Glibenclamide blocks K_ATP_ channels but does not affect dilation to the specific K_Ca_ channel activator NS1619 (Fig. 2). However, the selective K_Ca_ channel blocker paxilline, that does not inhibit dilation to pinacidil, fully blocks cerebral arteriolar dilation to NS1619 (Fig. 9). Also, paxilline attenuates dilation to hypercapnia and paxillin with glibenclamide abolish dilation of newborn pial arterioles in vivo (Fig. 10). Neither the magnitude of the dilatory response to hypercapnia nor these responses to paxillin and glibenclamide are affected by gender (Fig. 10).

## Discussion

The findings in this study of newborn pigs include: (1) in the newborn brain, smaller pial arterioles respond more robustly to hypercapnia than do larger pial arterioles; (2) glibenclamide, that effectively and selectively inhibits K_ATP_ channels, reduces but does not completely block dilation to hypercapnia; (3) paxillin, that effectively and selectively inhibits K_Ca_ channels, reduces but does not completely block dilation to hypercapnia; and (4) treatment with both glibenclamide and paxillin completely abolishes hypercapnia‐induced dilation. Overall, these data suggest hypercapnia activates both K_ATP_ and K_Ca_ channels leading to cerebral arteriolar dilation in newborn pigs.

K_ATP_ channels are among a group of interrelated mechanisms potentially determining tone in the cerebral arteries and arterioles (Gollasch et al. 1995; Zhuo et al. 2005). K_ATP_ channels are constructed of Kir6.0‐type subunits and sulfonylurea receptors and are inhibited by ATP (Zingman et al. 2002). These channels are sensitive to sulfonylureas and selective pharmacological channel openers, such as pinacidil (Huang and Chan 1998; Zhuo et al. 2005).

In the present report, in vivo, we demonstrated selective inhibition of brain surface arteriole K_ATP_ channels by topical application of glibenclamide. Glibenclamide at a dose of 10^−7^ mol/L blocked pial arteriolar dilation to the selective activator of K_ATP_ channels, pinacidil (10^−5^ mol/L) (Fig. 1). Here, we also show that glibenclamide does not affect control pial arteriolar diameter or dilation to NS1619 or isoproterenol (Fig. 2). We show that glibenclamide can partially prevent and partially reverse dilation to hypercapnia. When glibenclamide (10^−7^ mol/L) was injected onto the brain after 5 min of hypercapnia, it caused a 35% decrease in pial arteriolar diameter. Also, when the brain surface was pretreated with glibenclamide prior to the induction of hypercapnia, there was an ~60% decrease in pial arteriolar dilation compared to control without K_ATP_ channel inhibition.

The data in this report show that K_ATP_ channels are among the components that contribute to the dilation of pial arterioles to hypercapnia in newborn pigs. These data clearly demonstrate that glibenclamide inhibits pial arteriolar dilation to hypercapnia rather than totally blocking it. It is unlikely that the incomplete block of dilation to hypercapnia results from too little glibenclamide because 10^−7^ mol/L blocked dilation to pinacidil completely and 10^−6^ mol/L glibenclamide inhibited dilation to hypercapnia to the same degree as did 10^−7^ mol/L glibenclamide.

Our present data show that an additive relationship between K_ATP_ and K_Ca_ channels is sufficient to account for dilations of neonatal pial arterioles in vivo. Others have found that K_ATP_ channels are involved, to various degrees, in dilation of arteries and/or arterioles to hypercapnia (Lindauer et al. 2003; Phillis 2004; Phillis et al. 2004). Our finding that K_ATP_ channel blockade only partially inhibited dilation to hypercapnia in the newborn piglet arouses the question of what is responsible for the rest of the response. Because we showed in piglets that K_ATP_ and K_Ca_ channels are both involved in dilation to H_2_S and H_2_S is involved in dilation to hypercapnia (Leffler et al. 2011; Liang et al. 2011, 2012), it was logical to hypothesize that K_Ca_ channels, as well as K_ATP_ channels, may cause dilation of newborn pial arterioles to hypercapnia. We now demonstrate that blockade of both K_ATP_ and K_Ca_ channels abolishes newborn pial arteriolar dilation to hypercapnia, a primary control mechanism of cerebral vascular distribution of blood to match metabolic rate.

In a recent publication (Leffler et al. 2011), we found K_ATP_ channels to be among the mechanisms by which H_2_S causes dilation (Leffler et al. 2011). We also discovered that inhibition of H_2_S production also inhibited pial arteriolar dilation to hypercapnia. H_2_S increased K_ATP_ channel currents in piglet, freshly isolated, cerebral, arterial, smooth muscle cells (Liang et al. 2011). Glibenclamide fully reversed pinacidil‐induced K^+^ currents and partially inhibited H_2_S‐induced K^+^ currents (Liang et al. 2011). In isolated, pressurized piglet cerebral arterioles, glibenclamide completely blocked dilation to pinacidil, but only inhibited about half of the dilation to H_2_S (Liang et al. 2011), quite similarly to the partial block of hypercapnia‐induced pial arteriolar dilation by glibenclamide observed in the present study. In the case of H_2_S‐induced dilation, much of the K_ATP_ channel‐independent dilation can be attributed to an H_2_S‐induced elevation of sarcoplasmic reticulum K^+^ concentration that increases Ca^2+^ sparks to increase transient K_Ca_ current frequency (Liang et al. 2012).

Data in the present report that glibenclamide inhibits dilation of piglet pial arterioles in vivo in response to hypercapnia is in contrast to our earlier report (Leffler et al. 1997). In the present study, the efficacy of glibenclamide and the dilation to hypercapnia were measured in the same arterioles. We believe that K_ATP_ channels may not have been effectively inhibited in the earlier paper (Leffler et al. 1997).

Brain Paco_2_‐pH is the primary acute regulator of cerebral blood flow. Arteriolar Paco_2_ should have a quicker action than arteriolar pH because movement of ions, H^+^ and 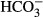, is restricted by the tight junctions of cerebrovascular endothelial cells that make up the blood–brain barrier (BBB). CO_2_ is highly water soluble and lipophilic, readily passing BBB. In fetus and newborn the neurovascular unit and its endothelial cells are particularly vulnerable to conditions injurious to the brain due to rapid proliferation and immature vascular structure. As a result, loss of endothelial function has been demonstrated to occur in the newborn as a consequence of ischemia–reperfusion, cerebral hemorrhage, and seizures. In piglets, seizures cause sufficient cerebral vascular endothelial injury to increase brain‐derived circulating endothelial cells (BCEC) over sevenfold (Parfenova et al. 2010). This increase mirrors loss of endothelial‐dependent relaxing factor function including the aforementioned hypercapnic pial artery dilation. Overall, alteration or injury to cerebral vasculature will cause neuronal injury as blood flow distribution does not match metabolism and the blood environment invades the neuropile.

In summary, our new data show there is a major contribution of K_ATP_ and K_Ca_ channels in the dilation of piglet pial arterioles to hypercapnia. This is, however, only part of the mechanism of CO_2_‐related vasodilation and more research is needed to draw conclusions on additional mechanisms.

## Acknowledgments

The content is solely the responsibility of the authors and does not necessarily represent the official views of the National Institutes of Health or the University of Tennessee.

## Conflict of Interest

None declared.
